# Editorial: Multi-omics and computational biology in horticultural plants: from genotype to phenotype, volume III

**DOI:** 10.3389/fpls.2025.1724809

**Published:** 2025-11-07

**Authors:** Yunpeng Cao, Mohammad Shah Jahan, Boping Wu, Lin Zhang

**Affiliations:** 1 Guangxi Colleges and Universities Key Laboratory for Cultivation and Utilization of Subtropical Forest Plantation, Guangxi Key Laboratory of Forest Ecology and Conservation, College of Forestry, Guangxi University, Nanning, China; 2 Key Laboratory of National Forestry and Grassland Administration on Cultivation of Fast-Growing Timber in Central South China, State Key Laboratory for Conservation and Utilization of Subtropical Agro-Bioresources, College of Forestry, Guangxi University, Nanning, China; 3 Department of Horticulture, Sher-e-Bangla Agricultural University, Dhaka, Bangladesh; 4 Collaborative Innovation Center for Efficient and Green Production of Agriculture in Mountainous Areas of Zhejiang Province, College of Horticulture Science, Zhejiang A&F University, Hangzhou, Zhejiang, China; 5 School of Basic Medical Sciences, Hubei University of Chinese Medicine, Wuhan, China

**Keywords:** multi-omics, computational biology, horticultural, genotype, phenotype

## Introduction

Horticultural plants underpin human nutrition, health, and well-being by providing essential vitamins, minerals, bioactive metabolites, and aesthetic values across fruits, vegetables, ornamentals, spices, and medicinal species (Lutaladio et al., 2010). Over the past decade, rapid advances in sequencing, phenotyping, and computational methods have transformed our capacity to interrogate the genetic and molecular bases of horticultural traits and to translate discoveries into breeding practice (Mansoor et al., 2025). Building on the momentum of our previous Research Topics—Volumes I and II—which together highlighted genome resources, integrative multi-omics, and emerging computational tools across a wide diversity of species (Mondal et al., 2022; Cao et al., 2024b)—this third volume continues the central mission: to bridge genotype and phenotype in horticultural plants through the integration of multi-omics with computational biology. Our goal with Volume III is twofold. First, to bring into focus integrative, cross-layered analyses that move beyond association toward mechanism and, ultimately, translatable targets. Second, to showcase methodological and infrastructural advances—ranging from single-cell and spatial omics to high-throughput phenotyping and interpretable machine learning—that strengthen the evidentiary chain linking genetic variation to complex traits under realistic environments (Großkinsky et al., 2015; Ferrão et al., 2023; Yan and Wang, 2023).

This Research Topic comprises two reviews and 14 research papers. The research papers include four on multi-omics (including transcriptomics, proteomics, and metabolomics) in horticultural crops, six on fruit crops, two on vegetables, one on spices, and one on model crop species.

## Advancing multi-omics integration in horticultural plants

Multi-omics integration combines genomic, epigenomic, transcriptomic, proteomic, and metabolomic data to provide a comprehensive view of biological systems. These contributions showcase how comprehensive molecular profiling, when combined with sophisticated analytical frameworks, enables unprecedented insights into complex trait formation in horticultural species. In this Research Topic, several studies demonstrate how combining multiple omics layers reveals regulatory networks and metabolic pathways that would remain hidden when analyzing single data types in isolation. For instance, Zhu et al. identified the molecular basis for color variations in *Cistanche deserticola*, showing that the purple hue of ‘oil cistanche’ stems from increased flavonoids and terpenoids, while its darker dried color is linked to higher levels of iridoids and polysaccharides. According to Tong et al., flavonoid biosynthesis in developing tobacco leaves shifts from synthesizing core structures in early growth, via upstream genes like *CHS* and *CHI*, to accumulating anthocyanins in later stages, driven by downstream genes like *DFR* and *ANS*. Lee et al. analyzed the responses of three hydroponic leafy vegetables to 24 stress conditions, creating the public database StressCoNekT to support breeding resistant crops and developing smart agriculture. Using multi-omics approaches, Wang et al. found that exogenous melatonin enhances salt tolerance in eggplant primarily by activating the α-linolenic acid metabolism pathway, while Gu et al. elucidated how melatonin priming modulates the waterlogging response in peach. Xuan et al. found that triploid hybrid jujube progeny exhibit significant horticultural advantages over their diploid counterparts, possessing typical polyploid characteristics such as wider leaves, larger stomata, longer thorns, and a significantly lower stomatal density. Chen et al. analyzed the chloroplast genomes of 35 *Rutaceae* species, providing a molecular framework for the family’s taxonomy and evolutionary history. These multi-dimensional analyses are particularly powerful for dissecting quantitative traits controlled by multiple genes and influenced by environmental factors.

## Multi-omics in horticultural plant breeding

In horticultural plants, such as tomato, strawberry, grape, apple, and peach, integrated datasets elucidate regulatory networks of ripening, color, flavor, texture, and nutrition; map stress−response pathways for heat, cold, drought, and salinity; and reveal disease−resistance mechanisms against major pathogens, while ionomics clarifies nutrient homeostasis and disorders such as calcium−related defects. Pangenomes and structural−variant catalogs expose presence–absence genes underlying quality and resilience traits, and haplotype−aware genomic prediction improves selection in perennials despite heterozygosity, clonality, and long juvenility. In grafted systems, multi−omics resolves rootstock–scion signaling that modulates vigor, nutrient uptake, stress tolerance, and fruit quality, and postharvest metabolomic and proteomic biomarkers guide shelf−life and cold−chain optimization. Single-cell and spatial transcriptomics have pinpointed key tissue-specific functions, such as sugar metabolism and flavonoid biosynthesis in the pericarp, embryo development and dormancy pathways in seeds, and cell division and differentiation programs in meristems, while rhizosphere and phyllosphere profiling has provided a basis for developing microbiome-informed strategies for biocontrol and nutrition (Deng et al.). Interoperable, FAIR databases (e.g., Sol Genomics Network, Genome Database for Rosaceae, Pear genomics database) harmonize data and ontologies to power germplasm discovery, marker−assisted and genomic selection, genome editing, and speed breeding—capabilities that are increasingly vital for sustaining quality, yield, and resilience under climate and resource constraints (Jung et al., 2007; Fernandez-Pozo et al., 2015; Chen et al., 2023).

## From discovery to application in horticultural plants

While maintaining strong foundations in basic research, Volume III emphasizes the translational aspects of multi-omics discoveries. Multiple studies demonstrate clear pathways from molecular insights to practical applications in crop improvement. For example, the identification of key genes controlling stress tolerance, nutritional quality, and yield components provides immediate targets for marker-assisted selection and genome editing approaches. In this Research Topic, studies on fruit and flower development revealed the regulatory role of the SlBEL11 factor in tomato ripening (Dong et al.) and identified the *CsAP2_51* gene as a direct regulator of gynostemium development in Chinese orchid (Wei et al.). Furthering work on developmental timing, Yu et al. conducted the first genome-wide analysis of the *FRI* gene family in soybean, where representative genes such as *GmFRI1* and *GmFRI2* are considered key regulators of flowering time through the modulation of FLOWERING LOCUS C (FLC)-like gene expression, consequently affecting crop adaptability to diverse environments. Other studies have likewise identified functionally important gene families. For instance, the *COMT*family in pear includes genes such as *PpCOMT1*, which plays a crucial role in the methylation of lignin biosynthesis intermediates, thereby influencing lignin content and fruit texture (Feng et al.). The *AHP* family, with representative genes like *MdAHP1* and *MdAHP3* in apple, acts as central positive regulators in the cytokinin signaling pathway, directly impacting cytokinin-mediated processes such as root and shoot development; *MdAHP3* has specifically been shown to negatively regulate adventitious root formation under cytokinin treatment (Li et al.). In addition, several analyses highlighted the roles of stress-responsive gene families. The *CDPK* gene family in jujube comprises members such as *ZjCDPK4* and *ZjCDPK11*, which are differentially expressed during fruit development, pathogen infection, and under abiotic stress conditions (Li et al.), indicating involvement in both developmental regulation and stress adaptation. The *SUS* gene family in blueberry features genes such as *VdSUS4*, which is upregulated under salt stress and has been demonstrated to improve salt tolerance in transgenic plants by facilitating sucrose metabolism, thus supporting energy supply and stress response (Wang et al.). Broadening this theme, Zhang et al. explored the molecular mechanisms by which WRKY transcription factors enhance plant stress resistance through their participation in sugar metabolism. These articles promote the broader use of multi-omics approaches in horticultural research and breeding.

## Emerging technologies and future directions in horticultural plants

Single-cell and spatial omics technologies are beginning to reveal cell-type-specific gene expression patterns and metabolic heterogeneity within plant tissues, providing unprecedented resolution for understanding developmental processes and stress responses. In this Research Topic, Deng et al. highlight that spatiotemporal transcriptomics enables precise mapping of gene expression dynamics across plant tissues, illuminating development, stress responses, and cell–cell communication *in situ*. Pan-genome analyses are uncovering structural variations and presence-absence polymorphisms that contribute to trait diversity but are missed by single reference genome approaches. Jiang et al. and Cao et al. conducted a comparative genomics study that identified novel lineage-specific new genes (including *de novo* gene and fusion gene), proposing them as a key mechanism for regulating plant growth and driving phenotypic evolution within the Rosaceae family (Cao et al., 2024a; Jiang et al., 2025). Ding et al. conducted a pear pangenome analysis and identified a SNP mutation and a promoter insertion in *PsbMGH3.1* that likely enhance sepal abscission in the ‘Xuehuali’ cultivar, a trait critical for fruit quality (Ding et al., 2024). Utilizing two newly assembled high-quality pear genomes for a genome-wide association study, Cao et al. identified and functionally validated the novel CCCH-type zinc finger gene *PbdsZF* as a key transcriptional regulator of lignin biosynthesis and stone cell formation, a critical determinant of fruit texture (Cao et al., 2025). In addition, the combination of biological big data with artificial intelligence is facilitating the transition from reactive to predictive and ultimately prescriptive approaches in crop management and improvement.

## Conclusion

Volume III of “Multi-omics and computational biology in horticultural plants: From genotype to phenotype” demonstrates the continued evolution and maturation of this interdisciplinary field. The 16 articles presented here showcase how integrative approaches combining multiple omics technologies are accelerating our understanding of complex traits in horticultural crops. Despite this remarkable progress, several challenges remain in fully realizing the potential of multi-omics approaches for horticultural crop improvement. Data integration across different omics layers and experimental conditions remains technically challenging, requiring sophisticated normalization and harmonization methods. Furthermore, the heterozygous and often polyploid nature of many horticultural crops presents unique challenges for genomic analyses and the functional validation of candidate genes. Developing robust analytical frameworks that account for this genetic complexity while maintaining computational efficiency remains an active area of research. Additionally, the translation of omics discoveries into field-relevant traits requires careful consideration of environmental variability and agricultural management practices. As we face mounting global challenges in food security, nutrition, and environmental sustainability, the approaches and discoveries presented in this Research Topic provide essential tools and knowledge for developing the next generation of improved horticultural varieties. The success of this Research Topic series reflects the growing recognition that bridging the genotype-phenotype gap requires not just more data, but smarter integration of diverse data types to address these very challenges. Looking forward, we anticipate that continued technological innovations, particularly in single-cell omics, spatial biology, and artificial intelligence, will be pivotal in overcoming these obstacles and will further enhance our ability to decode and manipulate the molecular basis of horticultural traits.
